# Supplementary figures and images for: Availability of Nanopore sequences in the genome taxonomy for Vibrionaceae systematics: Rumoiensis clade species as a test case
Source: PeerJ. 2018 Jun 18;6:e5018. doi: 10.7717/peerj.5018 (PMC6011873; doi:10.7717/peerj.5018)

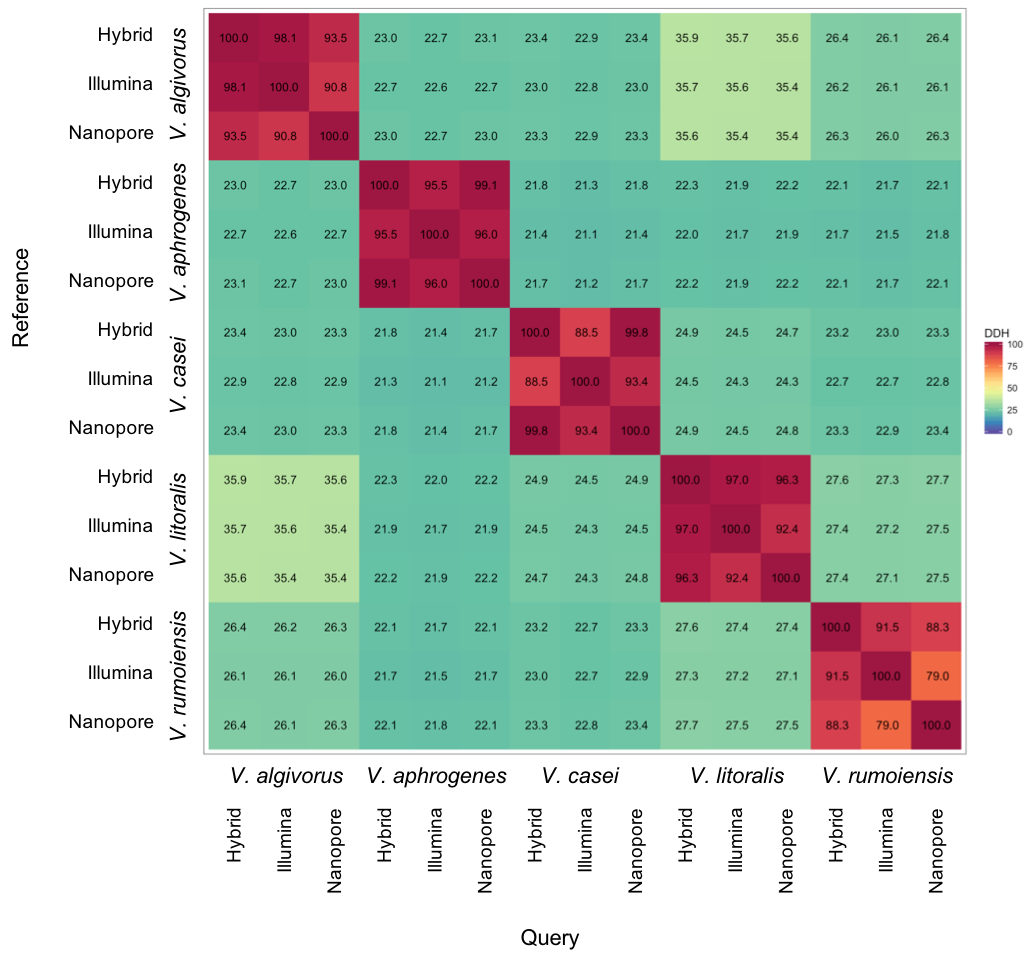

Supplement: Figure S1 — in silico DDH (DNA-DNA hybridization) values were estimated using Genome-to-Genome Distance Calculator (GGDC) 2.1 (Nelder & Wedderburn, 1972; Meier-Kolthoff et al., 2013) and the values represented here are calculated according to formula 2 (recommended). [file peerj-06-5018-s001.png]

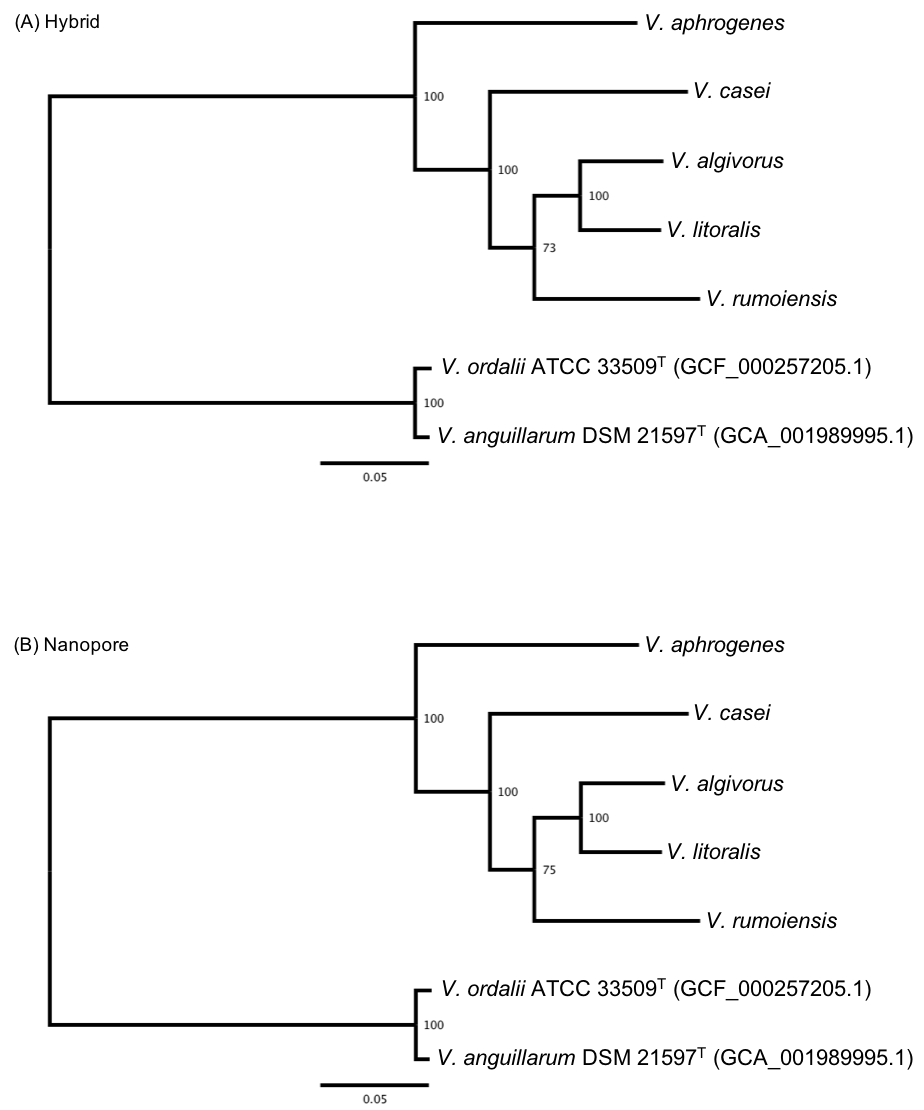

Supplement: Figrue S2 — Trees were reconstructed using RAxML 8.2.11 (Stamatakis, 2014) with the GTRGAMMA model and 500 bootstrap replications. The final trees were prepared using FigTree v1.4.3 (http://tree.bio.ed.ac.uk/software/figtree/). [file peerj-06-5018-s002.png]
