# Supplementary material for: Availability of Nanopore sequences in the genome taxonomy for Vibrionaceae systematics: Rumoiensis clade species as a test case
Source: PeerJ. 2018 Jun 18;6:e5018. doi: 10.7717/peerj.5018 (PMC6011873; doi:10.7717/peerj.5018)
Supplement: Table S1 — Basic information of the data used to assemble V. aphrogenes genome. The genome was sequenced in four different methods; SMRT system from Pacific Biosciences (PacBio), MinION from Oxford Nanopore Technologies (MinION), mate-pair (MiSeq-MP) and paired-end (MiSeq-PE) reads from Illumina MiSeq. For MinION, the data shown here is after debarcoding with Porechop 0.2.2 (https://github.com/rrwick/Porechop) with –untrimmed option. [file peerj-06-5018-s003.docx]

| Sequencer | PacBio | MinION | MiSeq-MP | MiSeq-PE |
| --- | --- | --- | --- | --- |
| Total reads | 45,370 | 42,584 | 2,387,353 × 2 | 2,442,013 × 2 |
| Total bases | 410,013,016 | 472,088,986 | 1,341,752,583 | 1,380,939,042 |
| Average length (bp) | 9,037 | 11,086 | 281 | 283 |
